# Supplementary material for: Efficient Separation of Photoexcited Charge at Interface between Pure CeO2 and Y3+-Doped CeO2 with Heterogonous Doping Structure for Photocatalytic Overall Water Splitting
Source: Materials (Basel). 2021 Jan 12;14(2):350. doi: 10.3390/ma14020350 (PMC7828182; doi:10.3390/ma14020350)
Supplement: Supplementary file 1 [file materials-14-00350-s001.pdf]

materials

MDPI

Supplementary Materials

Efficient Separation of Photoexcited Charge at Interface Between Pure CeO_2 and Y^{3+}-doped CeO_2 with Heterogonous Doping Structure for Photocatalytic Overall Water Splitting

Honghao Hou [1], Hirohisa Yamada [2], Atsumi Nitta [3], Yoshinori Murakami [4] and Nobuo Saito [1,*]

[1] Department of Materials Science and Technology, Nagaoka University of Technology, Nagaoka, Niigata 940-2188, Japan; wily9999@outlook.com
[2] Department of Chemical Engineering, National Institute of Technology Nara College, Nara 639-1080, Japan; yamada@nara.kosen-ac.jp
[3] Department of Environmental Materials, National Institute of Technology Niihama College, Niihama, Ehime 792-8580, Japan; anitta@mat.niihama-nct.ac.jp
[4] Department of Materials Engineering, National Institute of Technology Nagaoka College, Nagaoka, Niigata 940-8532, Japan; murakami_mb@nagaoka-ct.ac.jp
[*] Correspondence: saito@analysis.nagaokaut.ac.jp

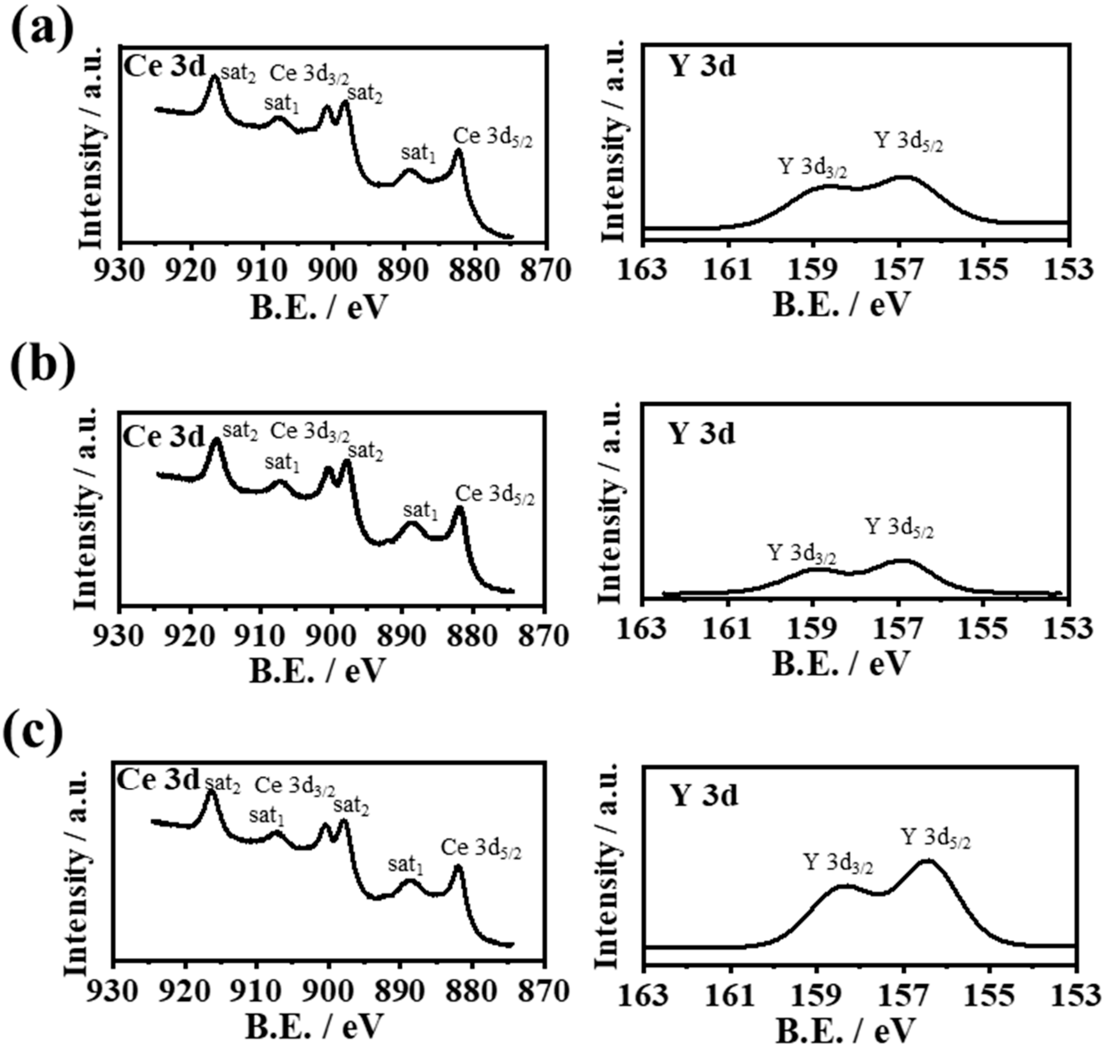

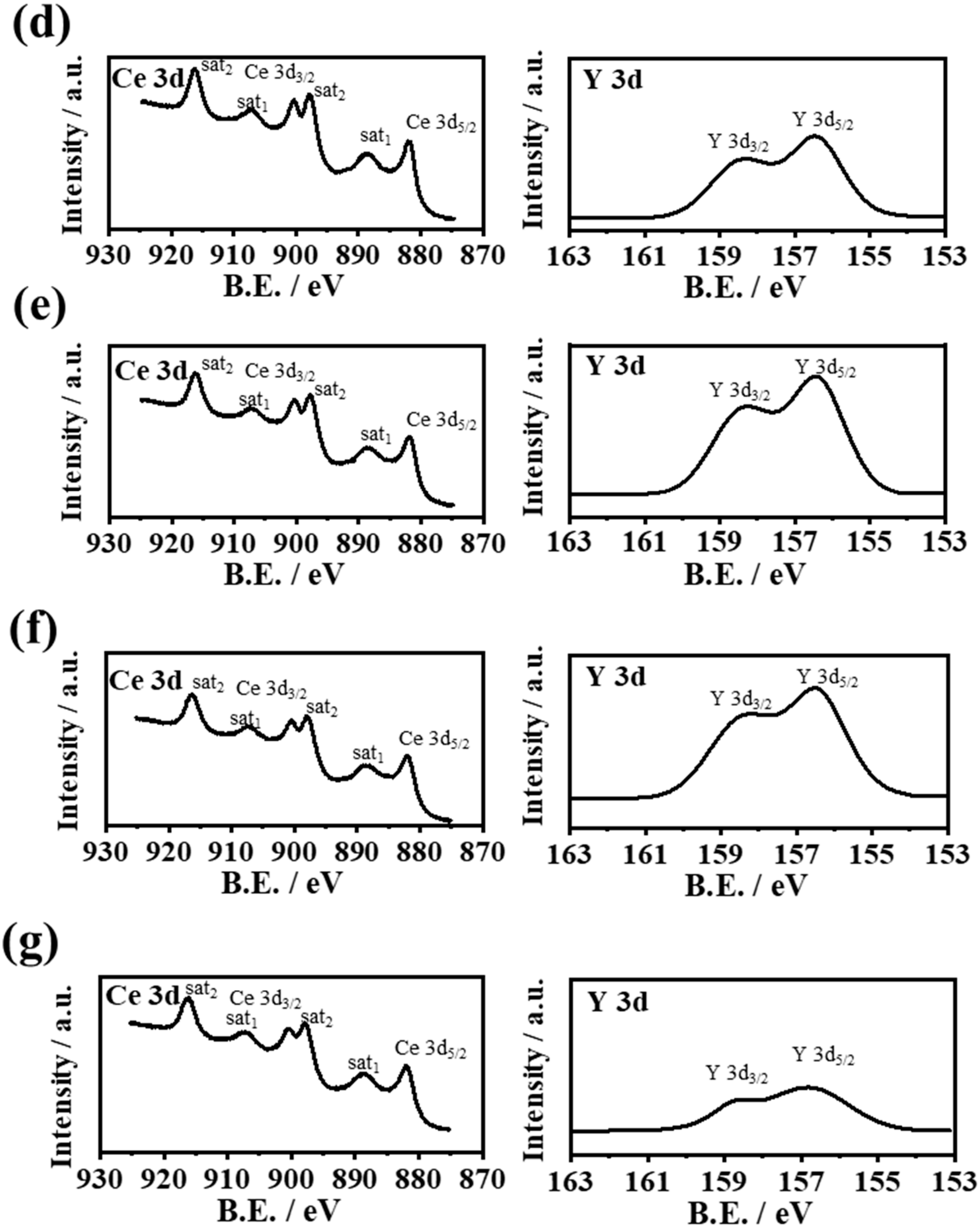

Figure 1. X-ray photoelectron spectra of Ce3d and Y3d for 1573K-CPT (a), 1273K-SSR (b), 1373K-SSR (c), 1473K-SSR (d),1573K-SSR (e),1673K-SSR (f), and 1773K-SSR (g).

For the oxidation state of Ce^{3+} and Ce^{4+}, the shape of Ce^{3+} in Ce 3d spectra is different with Ce^{4+}, in which characteristic peaks of Ce^{3+} appear in 880, 885, 899,904 eV, approximately [1]. In this study, no obvious appearance of Ce^{3+} peaks at 885, 880 eV in all XP spectrum were detected. Therefore, the oxidation states of Ce in all samples are probably tetravalent. These results are in good agreement with the XANES result reported by Matsui et al. [2]. In the case of Y 3d spectra, no obvious tendencies were observed with increasing doping temperature. Regarding the oxidation state of Y, Lee et al. [3] reported that the oxidation states of Y were trivalent by XANES after Y doping.

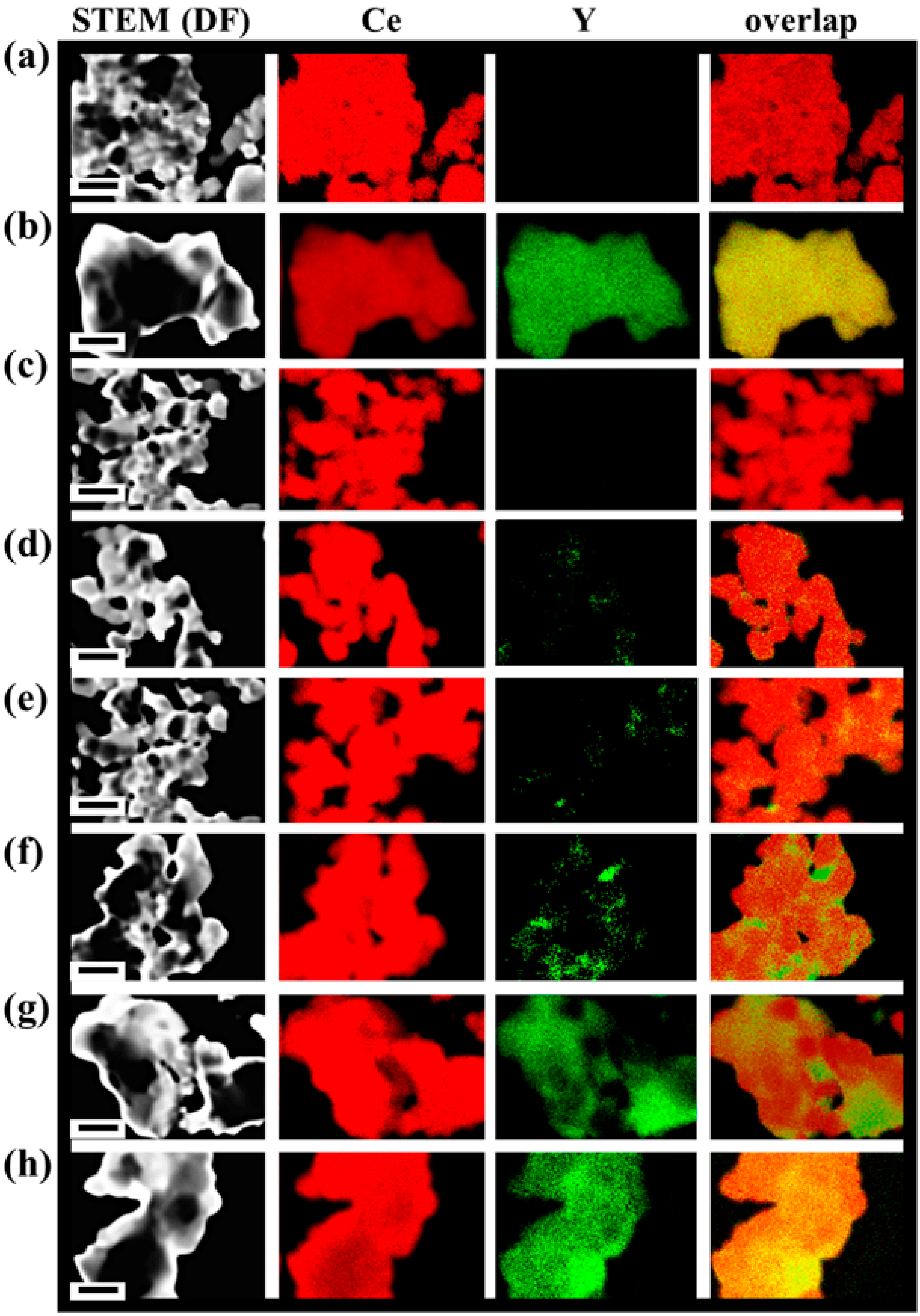

Figure 2. STEM/EDS mapping images of 1573K-Pure-CeO_2 (a), 1573K-CPT (b), 1273K-SSR (c), 1373K-SSR (d), 1473K-SSR (e), 1573K-SSR (f), 1673K-SSR (g), and 1773K-SSR (h). Scale bars are 400 nm.

Referense

[1]. Mullins, D. R.; Overbury, S. H.; Huntley, DR. Electron spectroscopy of single crystal and polycrystalline cerium oxide surfaces. *Surf. Sci.* **1998**, *409*, 307-319.

[2]. Yamazaki, S.; Matsui, T.; Ohashi, T.; Arita, Y. Defect structures in doped CeO_2 studied by using XAFS spectrometry. *Solid State Ionics* **2000**, *136*, 913-920.

[3]. Lee, W.; Chen, S. Y.; Chen, Y. S.; Dong, C. L.; Lin, H. J.; Chen, C. T.; Gloter, A. Defect structure guided room temperature ferromagnetism of Y-Doped CeO_2 Nanoparticles. *J. Phys. Chem. C* **2014**, *118*, 26359− 26367.
